# Sequence-specific cleavage of the RNA strand in DNA–RNA hybrids by the fusion of ribonuclease H with a zinc finger

**DOI:** 10.1093/nar/gks885

**Published:** 2012-10-05

**Authors:** Agata A. Sulej, Irina Tuszynska, Krzysztof J. Skowronek, Marcin Nowotny, Janusz M. Bujnicki

**Affiliations:** ^1^Laboratory of Bioinformatics and Protein Engineering and ^2^Laboratory of Protein Structure, International Institute of Molecular and Cell Biology in Warsaw, Ks. Trojdena Street 4, 02-109 Warsaw, Poland

## Abstract

Ribonucleases (RNases) are valuable tools applied in the analysis of RNA sequence, structure and function. Their substrate specificity is limited to recognition of single bases or distinct secondary structures in the substrate. Currently, there are no RNases available for purely sequence-dependent fragmentation of RNA. Here, we report the development of a new enzyme that cleaves the RNA strand in DNA–RNA hybrids 5 nt from a nonanucleotide recognition sequence. The enzyme was constructed by fusing two functionally independent domains, a RNase HI, that hydrolyzes RNA in DNA–RNA hybrids in processive and sequence-independent manner, and a zinc finger that recognizes a sequence in DNA–RNA hybrids. The optimization of the fusion enzyme’s specificity was guided by a structural model of the protein-substrate complex and involved a number of steps, including site-directed mutagenesis of the RNase moiety and optimization of the interdomain linker length. Methods for engineering zinc finger domains with new sequence specificities are readily available, making it feasible to acquire a library of RNases that recognize and cleave a variety of sequences, much like the commercially available assortment of restriction enzymes. Potentially, zinc finger-RNase HI fusions may, in addition to *in vitro* applications, be used *in vivo* for targeted RNA degradation.

## INTRODUCTION

Our understanding of the importance of RNA molecules in many life processes has changed dramatically in recent years. There is, however, much more to be discovered in this field. The application of modern analytical techniques to investigate the structure and function of RNA is not always possible due to the length of the transcript. Thus, the first step of analysis often involves the fragmentation of the RNA molecule(s) under study (1). Ribonucleases (RNases) are commonly used to carry out the nucleolytic cleavage of RNA into discrete fragments. However unlike, e.g., Type II restriction endonucleases (REases) that cleave DNA with high specificity toward particular sequences (2), most RNases are inherently non-specific. Those RNases that do exhibit specificity typically recognize either single bases or particular secondary structures in the RNA substrate such as helices, loops or bulges (3). The procurement of new enzymes that are able to cleave RNA in a purely sequence-dependent manner, similar to REases, would greatly facilitate studies on the structure and function of RNA and manipulation of these molecules for various purposes.

RNase HI is an enzyme that degrades the RNA strand of double-stranded DNA–RNA hybrids (4). It requires at least four continuous ribonucleotides in the substrate for cleavage (5). The mechanism for this substrate preference has been elucidated from the crystal structure of RNase HI from *Bacillus halodurans* in complex with a DNA–RNA hybrid. This enzyme recognizes a mixed A and B form conformation of the substrate (6). Several RNase HI amino acid residues make contacts with bases in the minor and major groove of the DNA–RNA hybrid, resulting in a very weak preference for substrates with a particular nucleotide sequence. Nonetheless, RNase HI and other related enzymes, must be considered inherently sequence non-specific.

The oligonucleotide-targeted cleavage method uses the non-specific RNase HI for the sequence/structure-specific fragmentation of RNA, e.g. for RNA structure probing (7), analysis of ribonucleoprotein complexes (8) and detection of microorganisms (9). In this method, a short DNA oligonucleotide is annealed to a longer single-stranded RNA molecule and used to direct cleavage of the RNA strand in the formed hybrid (5). Precise cleavage of a single, defined bond can be obtained by the usage of 2′-O-methyl RNA/DNA chimeras (10). The chimera is a single-stranded nucleic acid composed of a stretch of four deoxyribonucleotides flanked by methylated ribonucleotides. 2′-O-modified RNA hybridized to RNA is not recognized by the RNase HI as a substrate. However, the stretch of four deoxyribonucleotides forms a proper DNA–RNA hybrid and thus enables the cleavage of the RNA strand by the enzyme. The degradation of the RNA strand is thus limited to the region complementary to the DNA part of the chimeric oligonucleotide. Sequence-specific cleavage of RNA was also achieved through covalently linking RNase HI from *Escherichia coli* to a DNA oligonucleotide (11). Such conjugates have been demonstrated to be able to cleave specifically viral mRNA *in vitro* and *in vivo* (12,13). A DNA-fused RNase HI cleaves RNA at the region where its DNA adduct forms a DNA–RNA hybrid. A major cleavage site occurs in the middle of the complementary region and some secondary sites appear 1–3 nt away. A considerable disadvantage of employing conjugates is the necessity of chemical linking of the enzyme to the oligonucleotide.

RNase HI is not the only enzyme that is able to cleave RNA in DNA–RNA hybrids. Such an activity was demonstrated for a few REases (14). These enzymes were found to cut both DNA and RNA strands of the substrate in the recognition site, and the position of the scissile phosphodiester bond in the RNA strand of the DNA–RNA hybrid was the same as in the double-stranded DNA site. The rate of RNA strand cleavage was slower than of the DNA strand when uracil was present in the vicinity of the cleaved bond. The authors speculated that this activity may be attributed to these enzymes’ ability to bind A-form DNA or to induce conformational changes upon DNA binding toward the A-like form. It must be emphasized that the cleavage of DNA–RNA hybrids is a very rare feature among REases.

Besides naturally occurring REases that are able to cleave DNA–RNA hybrids, an enzyme composed of the catalytic domain of another REase FokI and an artificial zinc finger ZfQQR was engineered (15). That enzyme cleaved the DNA strand of the hybrid 3 nt away from the binding site, leaving the RNA intact. Zinc finger domains represent attractive protein modules for construction of fusion enzymes such as nucleases (16,17), transcription factors (18), methyltransferases (19), recombinases (20) etc. with tailored sequence specificity. They can be linked together in arrays in order to construct a binding domain that recognizes virtually any target sequence (21). Currently, zinc fingers are being replaced as DNA recognition modules by more specific transcription activator-like proteins (22–25) however, there are no reports that these domains are able to bind any other nucleic acid besides double-stranded DNA.

Here, we present an alternative approach of constructing a sequence-specific RNase H that cleaves a specific site in the RNA strand of the DNA–RNA hybrid, by fusing an engineered variant of the catalytic domain with a zinc finger that recognizes a specific sequence in the DNA–RNA hybrid.

## MATERIALS AND METHODS

### Gene cloning, protein expression and purification

The *rnhA* gene (NCBI accession number NC 002570) from *B. halodurans* was amplified from the pET15-rnhA plasmid (6) in a PCR reaction and cloned into the pET30b vector (Novagen) as a NdeI-KpnI fragment. The resulting construct, pET-30rnhA, was used to express a full-length RNase HI with a C-terminally fused hexahistidine peptide. The pET30-rnhAcat, which encoded the catalytic domain of RNase HI with a C-terminal hexahistidine peptide, was generated in a PCR reaction by removal of the fragment encoding residues 2-58 from the pET30-rnhA. The *zfqqr* gene was synthetized (Epoch Biolabs, protein sequence (26)) and cloned into pET28b vector (Novagen) as a NcoI-XhoI fragment. The pET-rnhAcatzfqqr construct was generated by ligation of fragments encoding residues 59-196 of the RNase HI and residues 7-107 of ZfQQR with a C-terminal fusion of a hexahistidine peptide. The pET-rnhAcatAEAzfqqr construct, which encoded a fusion protein variant with substitutions K138A, K146E, K180A (numbering according to amino acid sequence of the full-length RNase HI) in the RNase HI domain, was generated by sequential introduction of substitutions through PCR on the pET-rnhAcatzfqqr template. The pET-rnhAGQ and pET-rnhAGGKKQ variants (protein sequence in Supplementary Table S2) were generated by PCR-mediated deletion of fragments encoding amino acids 138-148 and 138-146 (a single glycine was introduced in place of the 138-146 deletion), respectively, from the pET-rnhAcatAEAzfqqr construct. All constructs were confirmed by DNA sequencing.

The *E. coli* strain ER2566 (New England Biolabs) was used for expression of the His-tagged proteins. Expression was induced with 1 mM IPTG for 5 h at 37°C, the cells were lysed in the continuous cell disruptor (Constant Systems LTD) at 20 000 psi and purified on His-Select Nickiel Affinity Gel (Sigma Aldrich). The lysis buffer contained 50 mM HEPES-KOH pH 8.0, 300 mM NaCl, 10 mM imidazole, 10% glycerol, 10 mM 2-mercapoethanol, 1 mM phenylmethylsulfonyl fluoride. The lysate was clarified by centrifugation at 40 000*g* for 30 min at 4°C. After 1 h incubation at 4°C with the clarified lysate, the resin was washed with 10 bed volumes of wash buffer 1 (lysis buffer with 2 M NaCl), 5 bed volumes of wash buffer 2 (lysis buffer with 20 mM imidazole) and the proteins were eluted with elution buffer (lysis buffer with 250 mM imidazole). The buffer was then exchanged using diafiltration to 25 mM Tris–HCl pH 8.0, 100 mM KCl, 10% glycerol, 2 mM DTT and samples were frozen at −70°C. The protein samples were run in 15% SDS-PAGE to asses purity and homogeneity was estimated at >90% (Supplementary Figure S1).

### Activity assay

The oligonucleotide RNA and molecular weight marker (Affymetrics) were 5′-labeled with [γ-33 P]ATP and T4 polynucleotide kinase (Ferments). The labeled substrate ssRNAs were gel purified. The DNA–RNA hybrid duplexes were formed by annealing the complementary ssDNA oligonucleotide in 10 mM Tris pH 6.5, 50 mM KCl, 10 µM EDTA. The sequence of the H1 substrate ssDNA: GGCTTGGGGAAGAACCGCCACGGTATCGATAAGCTTGATATCGAATTCCTGCAGCCCGGTTGATCCACTAGTTCT.

Activity assays were performed with 50 nM unlabeled and ∼2.5 nM end-labeled substrate in 25 mM Tris–HCl pH 8.0, 100 mM KCl, ZnSO_4_ and/or MgCl_2_ (concentrations indicated in descriptions to figures), 2 mM DTT. Reactions were carried out for 30 min or 1 h at 37°C and stopped by adding equal volume of formamide. Alkaline hydrolysis and T1 RNase (Fermentas) cleavage was done according to Ambion Life Technology’s recommendations. The samples were resolved on a 12% polyacrylamide/6 M urea gel and visualized by PhosphorImaging (Typhoon Trio, GE Healthcare). The gel for cleavage site mapping was vacuum dried before exposure to the Storage Phosphor Screen (GE Healthcare). All measurements of bands intensities were done using the ImageQuantTL software (GE Healthcare).

### Structural model

A structural model of the ZfQQR protein was created by homology modeling with SwissModel (27), using the crystal structure of a closely related designed zinc finger protein bound to DNA (PDB code 1mey) as a template (28). The DNA–RNA hybrid with the ZfQQR sequence corresponding to the H3 substrate was modeled with ModeRNA (29) and the interactions between the three zinc fingers domains and the nucleic acid were optimized by computational docking using the DARS-RNP potential (30). The model of the ZfQQR-nucleic acid complex was combined with the crystal structure of RNase HI from *B. halodurans* complexed with the DNA–RNA hybrid (PDB code 1zbi) (6). The final structure of the DNA–RNA hybrid in complex with the fusion protein was optimized using program Phenix (31). Amino acid substitutions in the RNase HI domain (K138A, K146E, K180A and N192D, numbering as in the original RNase HI sequence) and different variants of the interdomain linkers were introduced by homology modeling (modeled protein sequences in Supplementary Table S2).

## RESULTS

### Design and cleavage specificity of the fusion enzyme

RNase HI from *B. halodurans* comprises a hybrid binding domain (HBD) and the catalytic domain. The HBD binds to DNA–RNA hybrids in a sequence-independent manner and enhances the activity and processivity of the enzyme (32). An artificial zinc finger ZfQQR specifically binds the 5′-GGGGAAGAA-3′ sequence of the DNA strand in DNA–RNA hybrids (26). We constructed a fusion enzyme initially by connecting the catalytic domain of RNase HI (without the HBD) with ZfQQR using a 13 amino acid linker GRKGSGDPGKKKQ. The HBD was eliminated in order to lower the non-specific binding of substrates by the RNase HI moiety and to increase the overall sequence specificity of the fusion enzyme. This initial fusion enzyme is referred to as cat-ZfQQR. In order to further decrease the non-specific binding of the DNA–RNA hybrid by the RNase HI catalytic domain, we introduced substitutions of three Lys residues (K138, K146 and K180, numbering as in the original *B. halodurans* RNase HI sequence) that interact with phosphates of both strands of the substrate in the crystal structure of RNase HI in complex with a DNA–RNA hybrid (6). The substitutions K138A, K146E and K180A caused a significant decrease in the nucleolytic activity of the RNase HI catalytic domain lacking additional binding domains (Supplementary Figure S2). The enzyme variant with substitutions K138A, K146E and K180A is referred to as catAEA-ZfQQR.

**Figure 1.**
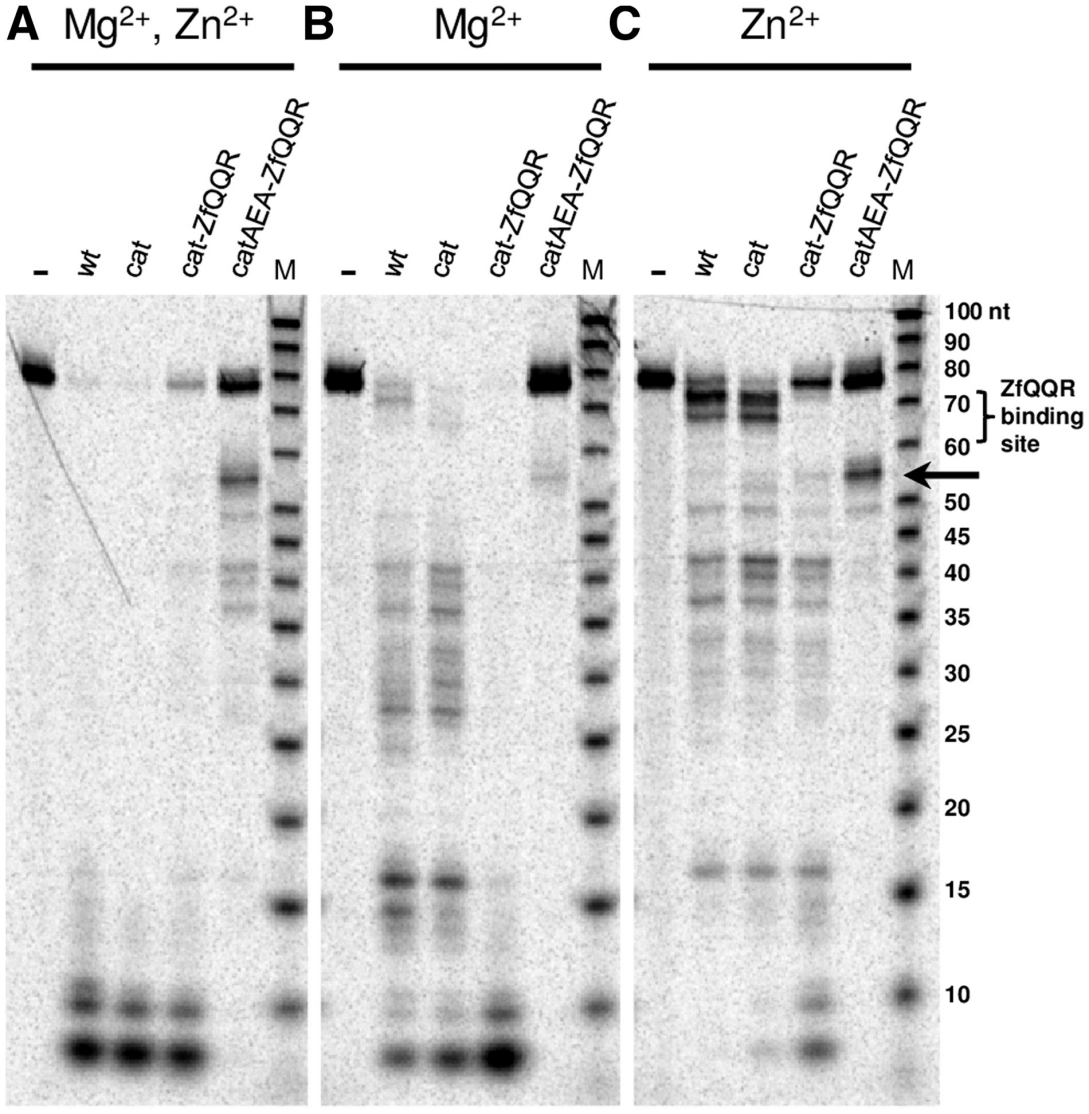
Cleavage of a DNA–RNA hybrid substrate H1 that contains the binding site for ZfQQR, in the presence of (**A**) 5 mM Mg^2+^ and 20 µM Zn^2+^, (**B**) only 5 mM Mg^2+^ and (**C**) only 20 µM Zn^2+^. The substrate H1 was incubated with RNase HI and fusion enzyme variants: 12.5 nM full-length *B. halodurans* RNase HI (wt), 625 nM catalytic domain of *B. halodurans* RNase HI (cat), 5 nM fusion of catalytic domain with ZfQQR (cat-ZfQQR), 25 nM fusion of catalytic domain with substitutions K81A, K89E and K123A with ZfQQR (catAEA-ZfQQR). A dash indicates that no enzyme was added. ‘M’ is the single-stranded RNA molecular mass standard. The bracket on the side of the RNA ladder marks the position of the ZfQQR binding site. The arrow indicates the position of the major cleavage product for the catAEA-ZfQQR variant.

The activity of the full-length RNase HI (wt), the catalytic domain of RNase HI (cat), and the fusion enzymes was assayed on a 75 bp DNA–RNA hybrid that contained the ZfQQR binding site (Figure 1). Mg^2+^ and Zn^2+^ were both included in the initial reaction conditions for the fusion enzyme, because RNase HI requires the presence of divalent metal ions for activity (6), and Zn^2+^ ions are crucial for the structural stability of the zinc finger. The cat-ZfQQR fusion enzyme cleaved rather indiscriminately in many positions along the entire RNA strand of the hybrid substrate, similarly to the wt RNase HI and to its isolated catalytic domain. However, the fusion enzyme with substitutions in the catalytic domain (catAEA-ZfQQR) exhibited a clearly distinct specificity, cleaving the RNA strand in close proximity of the ZfQQR binding site (between the 50th and 60th nt) and yielding a single major product (Figure 1).

We determined the effect of the presence of either Mg^2+^ and/or Zn^2+^ in the reaction on the specificity of the cleavage by catAEA-ZfQQR (Figure 1). The activity was highest in a buffer supplemented with both ions and lowest when only Mg^2+^ was present. The specificity of cleavage was highest when the reaction was performed with Zn^2+^ alone or when the concentration of Mg^2+^ was lowered to 50 µM (Supplementary Figure S3A and B). In all cases, however, non-specific cleavage products appeared in addition to the major one. Their presence could be attributed to some weak sequence-independent contacts between the enzyme and substrate or to the flexibility of the linker region between the two domains, which enabled the catalytic domain to cleave sites located at different distances from the ZfQQR binding site. In order to explore the first possibility, we supplemented the reaction buffer with heparin. Owing to its polyanion character and resemblance to the backbone structure of nucleic acids, heparin was shown to reduce binding of proteins to DNA and RNA (33). The addition of 0.5 µM heparin to the reactions with Mg^2+^ ions led to a complete inhibition of the fusion enzyme (Supplementary Figure S4), but in the Zn^2+^-containing buffer the inclusion of heparin resulted in an improvement of the cleavage specificity (Supplementary Figure S3C). The proportion of cleavage products resulting from the cleavage of the major site compared with non-specific sites cleavage improved 10-fold when the reaction was carried out with 20 µM Zn^2+^ and 6-fold in 50 µM. This improvement, however, had a considerable effect on the cleavage efficiency (we observed a 50% decrease of the reaction efficiency). We also made an attempt to destabilize the weak interactions with the substrate through introduction of additional substitutions in the substrate binding region of the catAEA domain variant. All five assayed variants N77A, N105A N106A, Q134A, R195A and R195E (numbering as in the original RNase HI sequence) had either greatly reduced activity, without change in the appearance of non-specific cleavage products, or were inactive (Supplementary Table S1). Because the attempt to increase the cleavage specificity of the cat-ZfQQR caused an overall decrease in the cleavage efficiency, we were not able to definitively conclude that the weak sequence-independent interactions were responsible for the non-specific activity of the variant. Thus, we decided to explore the second possible explanation and optimize the length of the interdomain linker.

### Model of the fusion enzyme and optimization of the linker length

**Figure 2.**
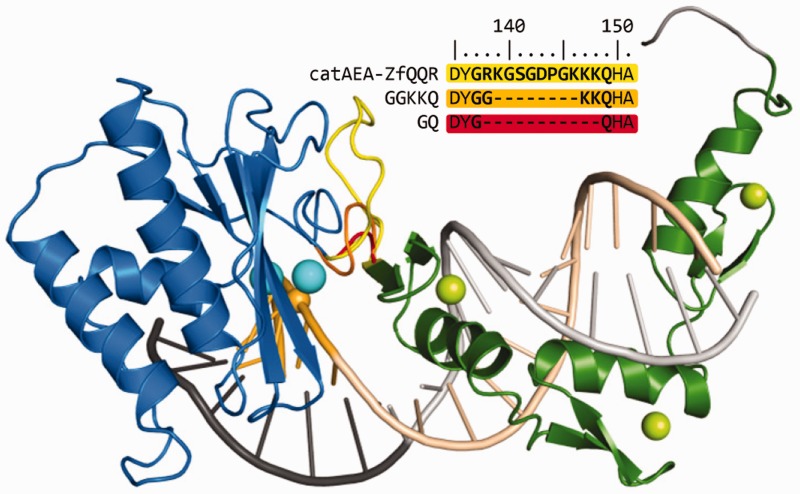
Structural model of the RNase HI-ZfQQR hybrid enzyme variants (catAEA-ZfQQR, GQ and GGKKQ) in complex with the DNA–RNA hybrid, with the cleavage site positioned 5 nt away from the ZfQQR binding site. Protein and nucleic acid backbone is shown in the cartoon representation: the RNase HI catalytic domain is shown in blue (Mg^2+^ ions are shown as cyan spheres), the ZfQQR module is in green (Zn^2+^ ions are shown as lime spheres), the DNA strand is shown in dark gray (with the sequence recognized by ZfQQR in light gray), and the RNA strand is in dark yellow (the sequence recognized by ZfQQR in light yellow). A phosphorus atom participating in the scissile phosphodiester bond is shown as a yellow sphere. The interdomain linkers of the catAEA-ZfQQR, GGKKQ and GQ variants are shown in yellow, orange and red, respectively. Sequences of the interdomain linkers for each variant are shown above the model.

To investigate the arrangement of the catalytic and substrate-binding domains in the fusion enzyme and to guide further engineering of the protein, we built a structural model of the catAEA-ZfQQR fusion enzyme complexed with the substrate (see Materials and Methods section for details). The catalytic domain was positioned to cleave in the most preferred location 5 nt away from the ZfQQR binding site (see Cleavage site mapping section for more details). The model (Figure 2) revealed that the initially used interdomain linker is longer than necessary and suggested that its shortening may decrease the cleavage at sites further away from the binding site. Based on the model, we designed two modifications, in which the interdomain linker was shortened by 11 and 8 amino acids, respectively. The resulting protein variants had only two (GQ) or five (GGKKQ) residues between the structurally important residue Y193 in the C-terminus of the RNase HI domain, and the N-terminal residue (H) of the first zinc finger domain in the ZfQQR module.

**Figure 3.**
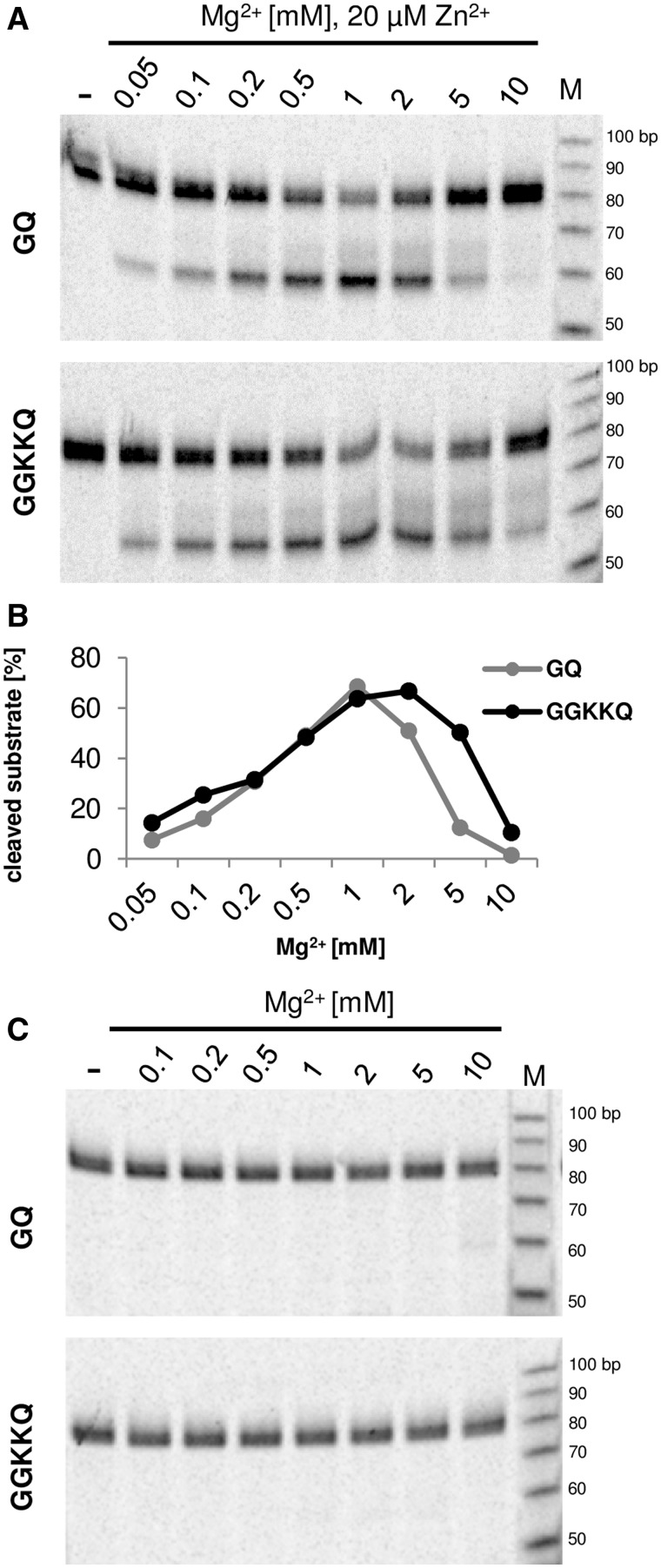
Cleavage of a DNA–RNA hybrid containing the binding site for ZfQQR by GQ and GGKKQ variants in different concentrations of ions (indicated above each lane). (**A**) The substrates were incubated with 50 nM of each enzyme in the presence of 20 µM Zn^2+^ and changing concentrations of Mg^2+^. A dash indicates that no enzyme was added. ‘M’ is the single-stranded RNA molecular mass standard. (**B**) A chart representing the fraction (in percentage) of product formed. 100% corresponds to the total intensity measured from each lane. (**C**) The substrates were incubated with 50 nM of each enzyme in the absence of Zn^2+^ and changing concentrations of Mg^2+^ as indicated above the lanes. A dash indicates that no enzyme was added. ‘M’ is the single-stranded RNA molecular mass standard.

We determined the effect of linker length reduction on the specificity of the substrate cleavage by GQ and GGKKQ variants. We tested a range of Mg^2+^ concentrations in the absence and presence of Zn^2+^ in the reaction (Figure 3). In case of both variants the cleavage was almost undetectable in the absence of Zn^2+^ (Figure 3A), however upon the addition of 20 µM Zn^2+^ the activity greatly increased (Figure 3B and C). For the GQ variant of the linker, the optimal concentration of Mg^2+^ in the reaction was 1 mM and for the GGKKQ variant it was 2 mM (Figure 3B). Different preferences with respect to the Mg^2+^ concentration were described earlier for variants of the human RNase H (34). In the case of both GQ and GGKKQ, concentrations of Mg^2+^ that were lower or higher than the optimal one, reduced the cleavage activity, a feature observed previously for wild-type RNase HI (16). We also tested the possibility of utilizing the RNase H fusion enzymes *in vivo* by performing a cleavage assay in a buffer that imitates physiological conditions (Supplementary Figure S5) and found that the GGKKQ variant cleaves the DNA–RNA hybrid substrate specifically and efficiently under these conditions.

### Cleavage site mapping

**Figure 4.**
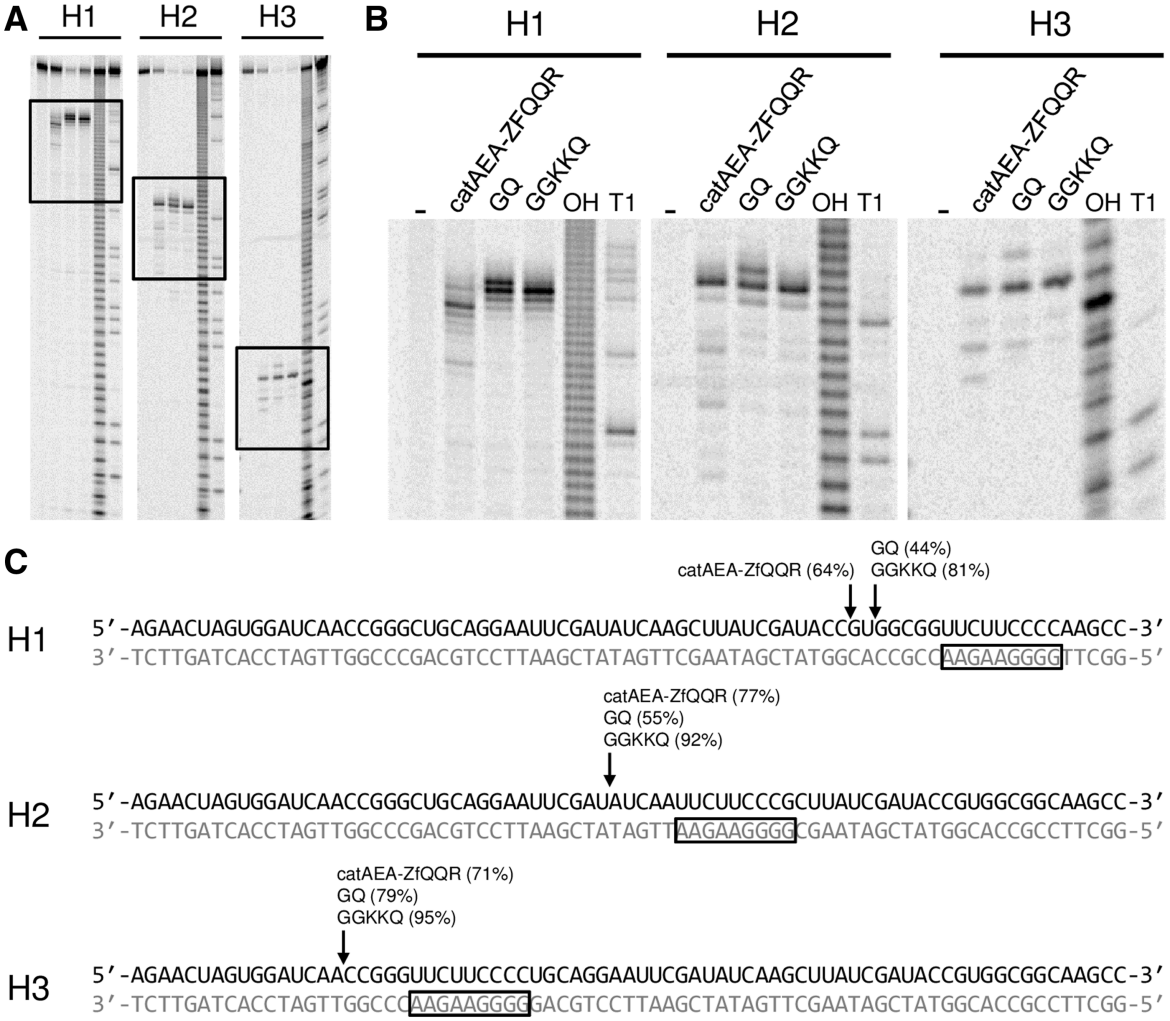
Cleavage site mapping on the H1, H2 and H3 DNA–RNA hybrids that contain a single-binding site for ZfQQR each. (**A**) Reactions were carried out in optimal conditions for each enzyme: 35 nM catAEA-ZfQQR in the presence of 20 µM Zn^2+^ and 0.5 µM heparin, 50 nM GQ in the presence of 20 µM Zn^2+^ and 1 mM Mg^2+^ and 50 nM GGKKQ in the presence of 20 µM Zn^2+^ and 2 mM Mg^2+^. A dash indicates that no enzyme was added. ‘OH’ indicates alkaline hydrolysis and ‘T1’ indicates RNase T1 cleavage of RNA. The rectangles mark the areas shown in panel (**B**). (**C**) Sequences of the three DNA–RNA hybrids. The main cleavage sites are indicated by arrows, the numbers above correspond to the percentage of cleavage at each location (100% is the total amount of product obtained after cleavage). The ZfQQR binding site is marked by a black box.

To confirm that the specific cleavage by catAEA-ZfQQR enzyme variants with GQ and GGKKQ linkers is solely dependent on the position of the binding site and is not influenced by the proximity of the physical end of the molecule or the nucleotide sequence around the cleavage site, we mapped the cleavage position in three different substrates (H1, H2 and H3). Substrates H2 and H3 were obtained by moving the binding site within the initial substrate H1 to two different positions, while leaving the rest of the sequence unchanged. Thus, the obtained substrates were of equal length, but had the ZfQQR binding site in different positions and flanked by different sequences. The cleavage of all substrates by the three enzyme variants occurred upstream of the recognition sequence and in its close proximity (Figure 4). All three substrates were cleaved by the GQ and GGKKQ variants mainly 5 nt upstream of the ZfQQR binding site. The shortening of the linker greatly improved the cleavage specificity, as it unified the distance of the major cleavage site with respect to the ZfQQR binding site across different substrates (the original catAEA-ZfQQR enzyme cleaved H1 in position 7 and H2 and H3 in position 5) as well as greatly reduced the amount of cleavage at additional sites. For none of the three fusion variants catAEA-ZfQQR, GQ or GGKKQ, cleavage downstream of the ZfQQR binding site could be detected. Some limited secondary cleavage could be observed upstream of the major cleavage site in the case of all enzymes. The GQ variant was found to cleave additionally 1 nt downstream of the major cleavage site, which can be attributed to the significantly reduced length of the linker (Supplementary Figures S6 and S7). The highest efficiency and specificity of cleavage under conditions of our assay was observed for the GGKKQ variant (Figure 4C and Supplementary Figure S6C). Measurements of kinetics of accumulation of the major and secondary cleavage products by the GQ and GGKKQ variants (Supplementary Figure S8) confirmed that the GGKKQ variant cleaves the substrate with higher specificity than the GQ variant. After 5 h incubation the secondary cleavage products constituted about 20% of the obtained product, whereas in the case of GQ it was almost 50%.

In addition to the cleavage experiments, binding assays were performed in order to determine the effect of the fusion of the RNase H domain to the zinc finger (Supplementary Figure S9). The fusion of catAEA to ZfQQR did not significantly affect the binding of the substrate, however the GQ and GGKKQ variants had binding reduced to about 40% in comparison to the ZfQQR alone. For all proteins, increased salt concentration (150 mM) lowered the binding by about 50% (Supplementary Figure S9A), which is consistent with reports on the salt dependence of DNA-binding by zinc finger proteins (35). We also measured the relative binding to a DNA–RNA hybrid that lacks the ZfQQR recognition site, and we found that all proteins, including ZfQQR alone, have the ability to bind such a nucleic acid non-specifically (Supplementary Figure S9B). The relative binding of the DNA–RNA hybrid with and without the ZfQQR site remains similar for the ZfQQR alone and for the ZfQQR-RNase H fusion proteins.

## DISCUSSION

Construction of enzymes with new specificities by fusion of various functional domains is a powerful technique. Here, we present engineering of a sequence-specific RNase HI, built from a processive sequence-independent enzyme RNase HI (32) and an artificial zinc finger. In order to obtain a fusion enzyme that cleaves the DNA–RNA hybrid in a position defined by the zinc finger specificity, we had to employ an approach that involved a number of optimization steps. The main obstacle we encountered was the control of sequence-independent cleavage by the catalytic domain of RNase HI.

The first optimization step of the fusion enzyme’s activity comprised modification of the RNase HI moiety by removal of the HBD and subsequently, by substituting positively charged residues K138, K146 and K180 involved in non-specific substrate-binding, by neutral (alanine) and negatively charged (glutamic acid) residues. Our aim was to minimize sequence-independent interactions between the catalytic domain and the nucleic acid, so that the substrate binding would be driven by ZfQQR moiety alone. The obtained catAEA-ZfQQR variant indeed cleaved in close proximity of the ZfQQR binding site, but the proportion between the major and non-specific cleavage products was unsatisfactory. The next optimization step involved varying the reaction conditions by changing buffer composition. Improved sequence-specific cleavage was obtained in conditions differing from those optimal for the catalytic activity of the wild-type RNase HI. The catAEA-ZfQQR variant cleaved optimally in the presence of Zn^2+^, but without Mg^2+,^ and when heparin was included. The positive effect of heparin on the cleavage specificity can most likely be attributed to at least partly selective disruption of the weaker electrostatic interactions between the non-specific sites and the fusion protein. In these conditions the sequence-independent cleavage by the fusion enzyme decreased, but the enzyme remained capable of cleaving the RNA strand in a sequence-dependent manner. Although the specificity increased, the optimization of reaction conditions did not give expected results in terms of cleavage efficiency.

The final step of improvement of the fusion enzyme specificity involved optimization of the linker between the binding and catalytic modules. We modeled the 3D structure of the fusion enzyme in complex with its substrate, to gain detailed insight into the positioning of the two domains on the substrate as well as into the possible flexibility of the linker. The variants obtained, GQ and GGKKQ, cleaved the RNA strand with specificity greatly increased in comparison to the catAEA-ZfQQR variant. The variant with the shortest linker (GQ) had a tendency to cleave 1 nt downstream of the major site, which was not observed for the other two enzymes. This is probably due to the short linker between the domains, which may hold the catalytic domain closer to the ZfQQR module than in the case of other variants, and cause it to cleave one residue closer. Unlike the catAEA-ZfQQR, the variants with optimized linker generate specific cleavage products in reactions with Mg^2+^ and without heparin, which are similar to the optimal conditions for the wild-type enzyme. These results supported our hypothesis that the flexibility of the linker between the domains was responsible for additional cleavage upstream of the major cleavage site when the substrate was treated with catAEA-ZfQQR. The results obtained in the binding experiments suggest that the fusion of RNase H domain affects the strength of binding the substrate when the interdomain linker is shortened, but we observed no effect of the fusion on the sequence specificity of the ZfQQR moiety.

Our results show that fusion of a RNase HI and a zinc finger permits the construction of a sequence-specific RNase H. Such an enzyme may potentially be utilized for *in vitro* manipulation of RNA and for *in vivo* studies on the process of genome instability caused by persistent DNA–RNA hybrids formed during transcription. Zinc fingers are relatively easy targets for specificity engineering, hence our fusion enzyme should be amenable to redesign toward construction of RNases H specific for custom sequences. Current RNA fragmentation protocols involve partial cleavage with a base-specific ribonuclease, treatment with ultrasounds or usage of a ribozyme. In case of the first two methods, the utilization of a sequence-specific RNase H will facilitate the control of the fragmentation procedure and allow to obtain molecules with defined ends. The advantage of using an enzyme fusion over ribozymes lies in the simplicity of separation of nucleic acids from the protein, as they differ in biochemical properties.

## SUPPLEMENTARY DATA

Supplementary Data are available at NAR Online: Supplementary Tables 1 and 2 and Supplementary Figures 1–9.

## FUNDING

The Polish Ministry of Science and Higher Education (MNiSW) [R12 002 02]; European Research Council (ERC) [StG RNA+P=123D]; Scholarship from the European Social Fund, Human Capital Operational Programme for the execution of the project ‘Support for bio tech med scientists in technology transfer’ [UDA-POKL.08.02.01-14-041/09 to A.A.S.]; Foundation for Polish Science (FNP) [START fellowship to I.T.]; International Early Career Scientist grant from the Howard Hughes Medical Institute (to M.N., in part); ‘Ideas for Poland’ fellowships from the FNP (to M.N. and J.M.B.). Funding for open access charge: ERC [StG RNA+P=123D].

*Conflict of interest statement*. None declared.

## Supplementary Material

Supplementary Data
