# Supplementary material for: Sequence-specific cleavage of the RNA strand in DNA–RNA hybrids by the fusion of ribonuclease H with a zinc finger
Source: Nucleic Acids Res. 2012 Oct 5;40(22):11563–70. doi: 10.1093/nar/gks885 (PMC3526281; doi:10.1093/nar/gks885)
Supplement: Supplementary Data [file supp_40_22_11563__index.html]

Sequence-specific cleavage of the RNA strand in DNA–RNA hybrids by the fusion of ribonuclease H with a zinc finger — Sequence-specific cleavage of the RNA strand in DNA–RNA hybrids by the fusion of ribonuclease H with a zinc finger — Supplementary Data 

# Sequence-specific cleavage of the RNA strand in DNA–RNA hybrids by the fusion of ribonuclease H with a zinc finger

## Supplementary Data

files

**Files in this Data Supplement:**

- Supplementary Data - pdf file
