# Supplementary material for: Sequence-specific cleavage of the RNA strand in DNA–RNA hybrids by the fusion of ribonuclease H with a zinc finger
Source: Nucleic Acids Res. 2012 Oct 5;40(22):11563–70. doi: 10.1093/nar/gks885 (PMC3526281; doi:10.1093/nar/gks885)
Supplement: Supplementary Data [file supp_gks885_nar-01645-f-2012-File006.pdf]

Supplementary materials

Fig. S1. Protein sample analysis on the 15% SDS-PAGE gel. The amount of purified protein in each lane corresponds to about 5 ml of bacterial culture after induction.

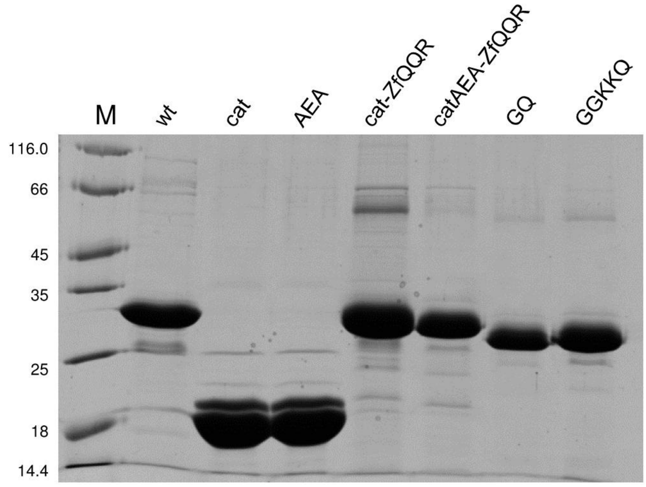

Fig. S2. Cleavage of a DNA-RNA hybrid substrate in the presence of 5 mM Mg^{2+}. The substrate H1 was incubated with a range of concentrations of RNase HI variants (indicated above each lane): (A) catalytic domain of *B. halodurans* RNase HI, (B) catalytic domain with substitutions K81A, K89E, and K123A. A dash indicates that no enzyme was added. "M" is the single-stranded RNA molecular mass standard.

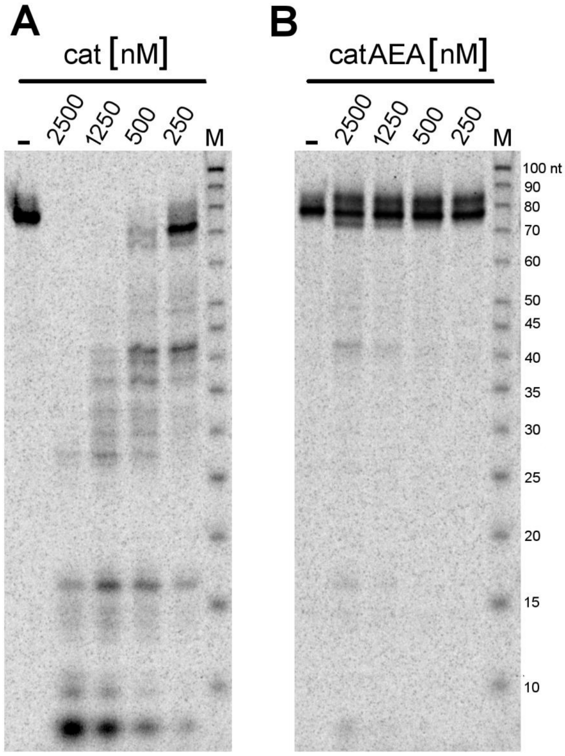

Fig. S3. Cleavage of a DNA-RNA hybrid substrate by catAEA-ZfQQR in different concentrations of ions (indicated above each lane): (**A**) Mg^{2+}, (**B**) Zn^{2+} and (**C**) Zn^{2+} with 0.5 µM heparin. Substrate H1 was incubated with 35 nM catAEA-ZfQQR. A dash indicates that no enzyme was added. "M" is the single-stranded RNA molecular mass standard. Below each audioradiograph is a chart that presents the dependence of the intensity of major product and secondary cleavage products on the concentration of ions in the reaction. 100% corresponds to the total intensity measured from each lane.

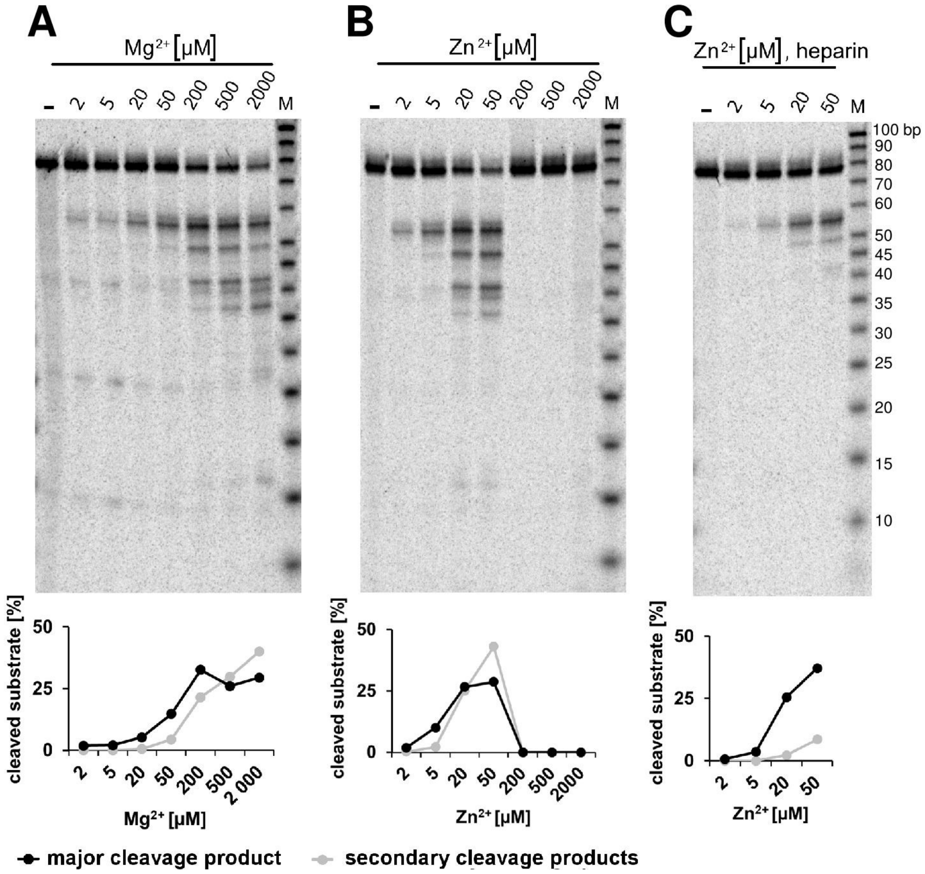

Fig. S4. Lack of cleavage of a DNA-RNA hybrid substrate that contains the binding site for ZfQQR by catAEA-ZfQQR in the presence of 0.5 μM heparin in the reaction. The substrate H1 was incubated with a 35 nM catAEA-ZfQQR in the presence of changing concentrations of Mg^{2+} as indicated above the lanes. Zn^{2+} was not included in the reaction. A dash denotes no enzyme was added. "M" is the single-stranded RNA molecular mass standard.

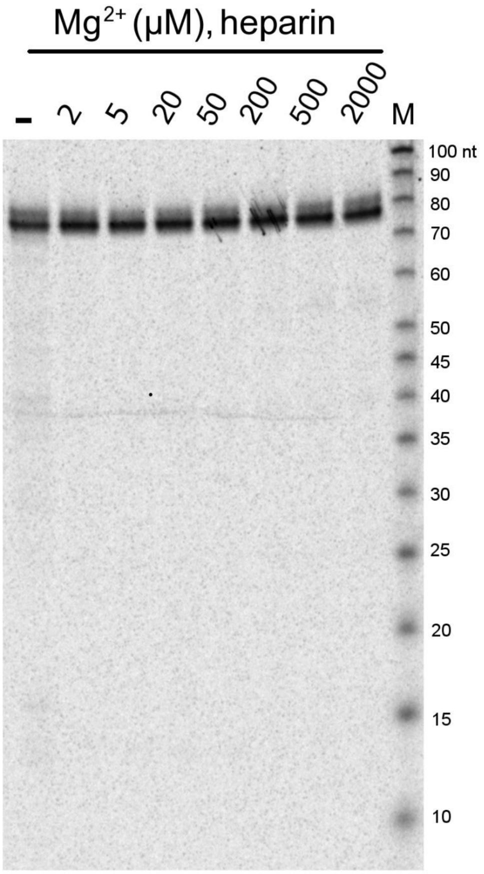

Fig. S5. Cleavage of a DNA-RNA hybrid substrate by GQ and GGKKQ under physiological conditions in different concentrations of spermidine (indicated above each lane). Activity assays were performed with 50 nM unlabeled and approximately 2.5 nM end-labeled substrate in 1 x PBS (10 mM Na_2HPO_4, 2 mM KH_2PO_4, 137 mM NaCl, 2.7 mM KCl, pH adjusted to 7.4 with HCl) supplemented with 0.8 mM $MgCl_2$, 2 µM $CaCl_2$, 7 µM $ZnSO_4$ and different concentrations of spermidine. Reactions were carried out for 3 hours at 37°C and stopped by adding equal volume of formamide. A dash denotes no enzyme was added. "M" is the single-stranded RNA molecular mass standard.

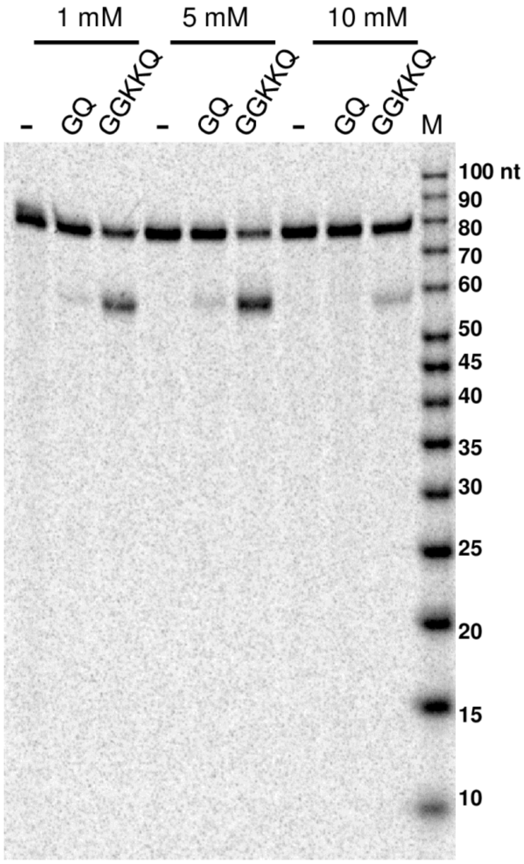

Fig. S6. Cleavage site mapping for (A) catAEA-ZfQQR, (B) GQ and (C) GGKKQ on DNA-RNA hybrid substrates H1, H2, and H3 that contain a binding site for ZfQQR. The cleavage sites are indicated above sequences of the three DNA-RNA hybrids by arrows, the numbers above correspond to the cleavage efficiency (in percent of the total product) at each location. The main cleavage site is marked with a large arrow and secondary sites are marked with small arrows. The ZfQQR binding site is marked by a black box.

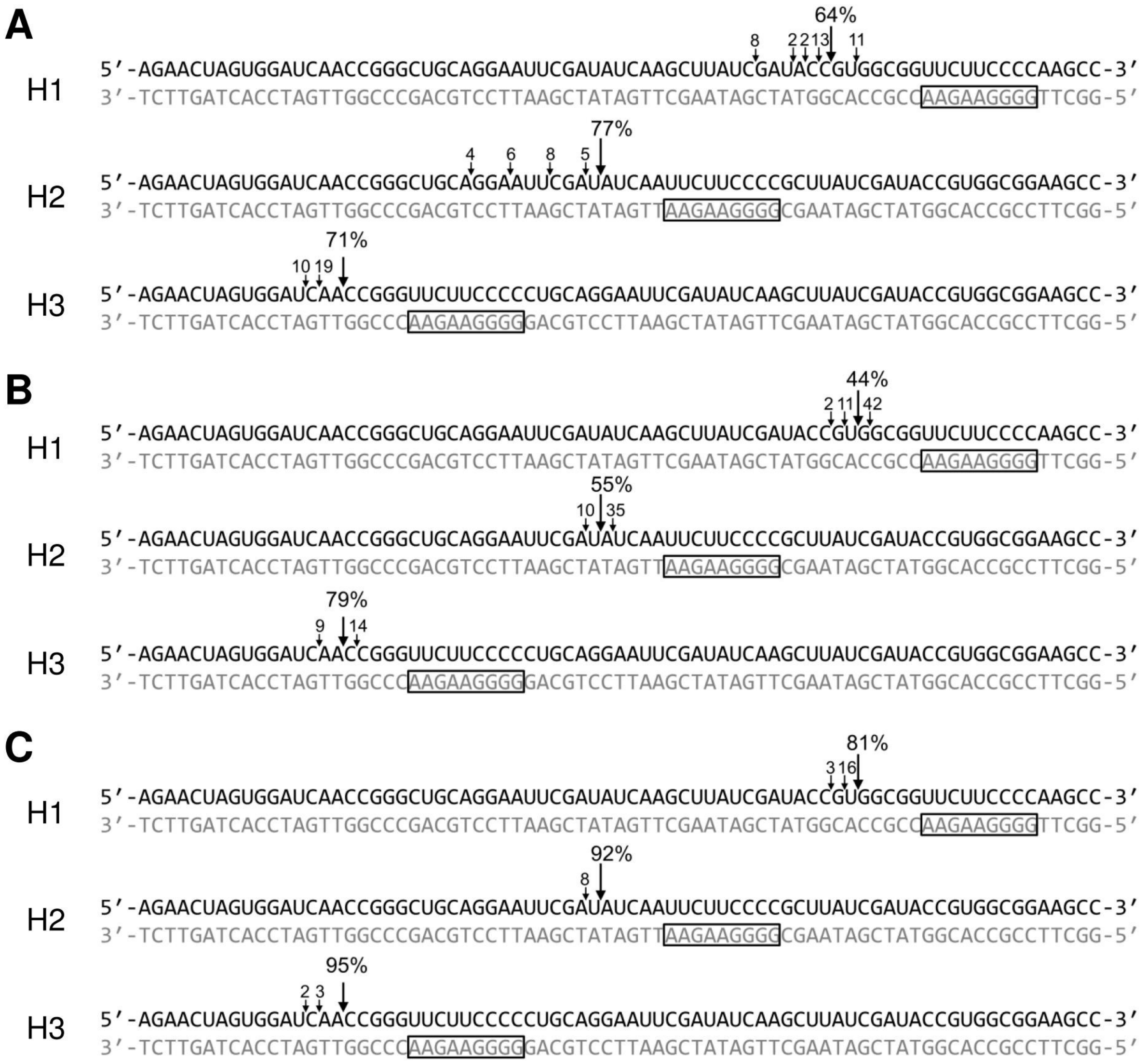

Fig. S7. Structural model of the RNase HI- ZfQQR hybrid enzyme with interdomain linker GQ in complex with the DNA-RNA hybrid, with the cleavage site positioned 4 nt away from the ZfQQR binding site**.** Protein and nucleic acid backbone is shown in the cartoon representation: the RNase HI catalytic domain is shown in blue (Mg^{2+} ions are shown as cyan spheres), the ZfQQR module is in green (Zn^{2+} ions are shown as lime spheres), the DNA strand is shown in dark grey (with the sequence recognized by ZfQQR in light grey), and the RNA strand is in dark yellow (the sequence recognized by ZfQQR in light yellow). A phosphorus atom participating in the scissile phosphodiester bond is shown as a yellow sphere. The interdomain linker GQ is shown in red.

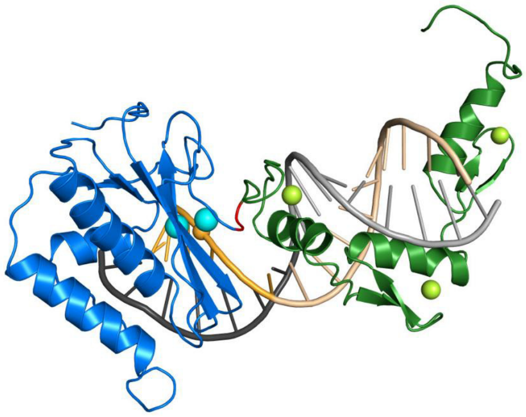

Fig. S8. Cleavage kinetics of a DNA-RNA hybrid substrate by (A) 20 nM GQ and (B) 40nM GGKKQ. The reactions were stopped after 15 min, 30 min, 1, 2, 3 and 5 hours by adding equal volume of formamide. The intensities of the major product and of all secondary products were measured and the percentage of the products formed was plotted on a chart. The reactions were repeated three times, the error bars show the standard deviation of these measurements. 100% corresponds to the total intensity (a sum of the intensities of the uncleaved substrate and the cleaved products) measured from each lane.

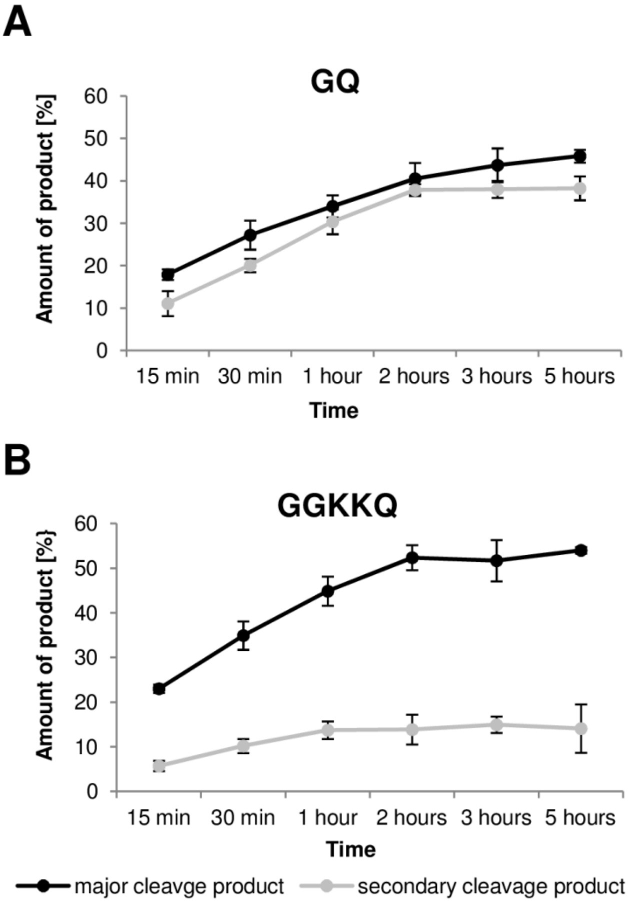

Fig. S9. Relative binding of the DNA-RNA hybrids by ZfQQR, catAEA-ZfQQR D192N, GQ D192N, and GGKKQ D192N variants, measured with the nitrocellulose filter binding assay. The D192N substitution (numbering as in the original RNase HI sequence) was introduced in order to obtain enzymatically inactive variants for the assay. An oligonucleotide with the ZfQQR binding site (TCACTGGGGAAGAAGAATCCTCGATATAGTTCCTCCTTTC) and with a non-cognate sequence (ACAATCCTGTCGTTACCTGTCATGTATCCGTCTAGAT) were radiolabelled with [γ-33P]ATP and T4 polynucleotide kinase, annealed to the complementary RNA oligonucleotide and purified on Sephadex G-25 to remove the unincorporated label. Enzyme-substrate mixtures (50 µl total volume) were incubated in the binding buffer (25 mM Tris-HCl pH 8.0, KCl (at concentration indicated on the figure), 1 mM $MgCl_2$, 20 µM $ZnSO_4$, 10 µg/ml BSA) containing a mixture of 1 nM of radiolabelled substrate and 100 nM unlabeled substrate and 1 µM protein. Reactions were incubated 30 min. at 25°C and filtered through 0.22 mm nitrocellulose filter (Whatman) in Dot-Blot apparatus (Bio-Rad). Each well was washed with 400 µl of the binding buffer. Dried filters were exposed to a phosphoimager screen overnight. Images were scanned on a Storm Phosphorimager, and the retained radioactivity was quantified using ImageQuant software (Amersham). For each protein triplicate measurements of binding were made and standard deviation was calculated. (A) Relative binding of the sequence GGGGAAGAA in different salt concentrations. 100% is the binding of ZfQQR to the substrate in 100 mM KCl. (B) Comparison of the relative binding of the GGGGAAGAA and non-cognate sequences. 100% is the binding of ZfQQR to GGGGAAGAA.

A

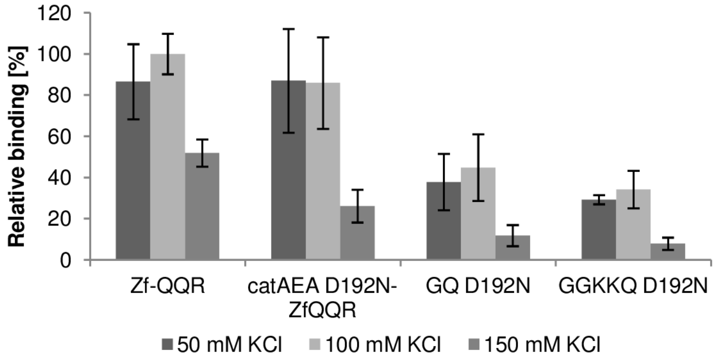

B

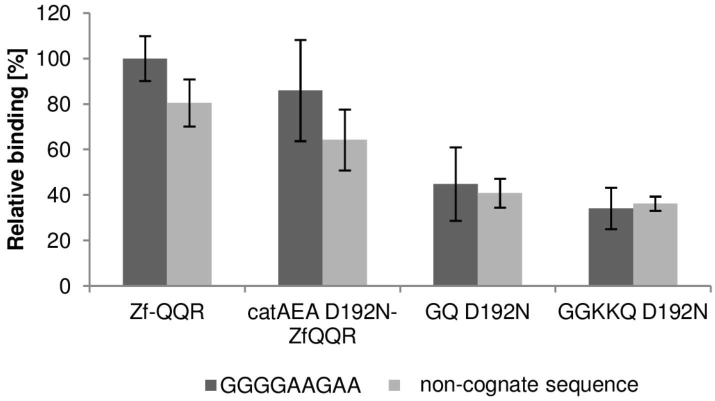

Table S1. The amino acid substitutions introduced in the catAEA-ZfQQR variant and their effect on activity.

Substitution numbering according to the full-length RNase HI	Substitution numbering according to the catAEA-ZfQQR	Role of aa in RNase HI *B. halodurans* (5)	Effect on activity
N77A	N20A	Interaction with DNA strand backbone and bases	Cleavage undetectable
N105A N106A	N48A N49A	Interaction with DNA strand backbone and bases	Cleavage undetectable
Q134A	Q77A	Interaction with RNA sugars	Cleavage undetectable
R195A	R138A	Sequence-specific contact in the crystal structure of RNase HI-hybrid complex	4 times lower activity
R195E	R138E	Sequence-specific contact in the crystal structure of RNase HI-hybrid complex	100 times lower activity

Table S2. The protein sequences of catAEA-ZfQQR, GQ and GGKKQ fusion protein variants. The linker region is shown in bold and the ZfQQR moiety is shown in italic.

Plasmid construct name	Protein name	Protein sequence
pET-rnhAcatAEAzfqqr	catAEA-ZfQQR	MAKEEIIWESLSVDVGSQGNPGIVEYKGVDTKTGEVLFEREPIPIGTNNMGEFLAIVHGLRYLKERNSRKPIYSDSQTAIAWVKDKKAASTLVRNEETALIWKLVDEAEEWLNTHTYETPILAWQTDKWGEIKADY**GRKGSGDPGKKKQ***HACPECGKSFSQSSNLQKHQRTHTGEKPYKCPECGKSFSQSSNLQKHQRTHTGEKPYKCPECGKSFSRSDHLQRHQRTHQNKKLE*HHHHHH
pET-rnhAGQ	GQ	MAKEEIIWESLSVDVGSQGNPGIVEYKGVDTKTGEVLFEREPIPIGTNNMGEFLAIVHGLRYLKERNSRKPIYSDSQTAIAWVKDKKAASTLVRNEETALIWKLVDEAEEWLNTHTYETPILAWQTDKWGEIKADY**GQ***HACPECGKSFSQSSNLQKHQRTHTGEKPYKCPECGKSFSQSSNLQKHQRTHTGEKPYKCPECGKSFSRSDHLQRHQRTHQNKKLE*HHHHHH
pET-rnhAGGKKQ	GGKKQ	MAKEEIIWESLSVDVGSQGNPGIVEYKGVDTKTGEVLFEREPIPIGTNNMGEFLAIVHGLRYLKERNSRKPIYSDSQTAIAWVKDKKAASTLVRNEETALIWKLVDEAEEWLNTHTYETPILAWQTDKWGEIKADY**GGKKQ***HACPECGKSFSQSSNLQKHQRTHTGEKPYKCPECGKSFSQSSNLQKHQRTHTGEKPYKCPECGKSFSRSDHLQRHQRTHQNKKLE*HHHHHH
